# Gun Shot Wounds

**Published:** 1876

**Authors:** D. S. Handy

**Affiliations:** Stephenville, Texas


### GUN SHOT WOUNDS.

BY D. S. HANDY, M.D., STEPHENVILLE, TEXAS.

On the 21st of November, 1874, I was called to see Charles Ellington, aged 23 years, dark complexion, steady habits, good health, and, by occupation, a farmer. Found him suffering from gun shot wounds, inflicted by Indians some three hours previous. A ball from a Winchester rifle (caliber 44–100), had entered the back in the vicinity of the fourth rib, two inches outside of the left scapula, and, ranging forward, emerged two and a half inches above the left nipple. A smaller shot (likely from a pistol) entered the right arm at the union of the middle and lower thirds, followed the course of the humerus to the shoulder, passed beneath the right scapula, and came out near the spinos processes of the vertebra. A flesh wound on the left forearm claimed but slight attention.

The symptoms, usually caused by penetrating wounds of the thorax, were present in a marked degree, viz: collapse, anxious countenance, difficult breathing, etc. He had lost much blood, and the air was passing in and out at the wound above the nipple, as he breathed. Pulse rapid and bounding, but soft; skin covered with perspiration. He was not scared, and bore the necessary probings with patience, insisting that he could sit up while the wounds on his back were dressed, but was compelled to lie down. Says the Indians were not more than five paces from him when they shot him. He walked a full half mile after he was wounded, but had to sit down and rest twice.

I immediately closed the wound with Robbin's Isinglass Plaster, thereby excluding the air, but not confining the drainage, as the liquid portion of the blood coming in contact with the plaster, would soften it and escape between it and the integument.

Ordered whisky, ℥ ii; fluid ext. ergot, ʒ i; morphia sul., ¼ grain every two hours.

November 22d.—Rests well—treatment continued.

November 23d.—Very restless; cough troublesome; delirious at night.

R. Morphia sulphas, grs. ii.
Pulv. Doveri, grs., xl.
Divide in pulv. viii.

Give one every two hours.

November 24th and 25th.—Very quiet, treatment continued.

November 26.—Secondary hemorrhage came on at 1 o'clock, P.M.; pulse, 90 beats per minute; perspires freely. Gave fluid ext. ergot, ʒ i; tinct. ver. vir., gtt. iii. every two hours.

6 o'clock, P. M.—Hemorrhage has ceased; pulse 65 beats per minute; complains of nausia.

Gave morphia sulphas, gr. ¼; whisky, two ounces every two hours.

10 o'clock, P.M. Sleeping quietly.

At this time he was coughing up great mouthfuls of blood, and continued to do so for some days. His appetite, though not good, was improving. A nourishing diet was given, and only enough pulv. opii to quiet the cough. A strong egg-nog was allowed almost *ad libitum.* He did not complain of the wound in the left forearm, or the one in the right forearm and shoulder. The lung healed kindly, and in January he was able to start on a trip of one hundred miles, and I lost sight of him, though I learn he is doing well. I might add, that where the balls entered, the wouud soon healed, but where they came out puss was discharged for some time.

The wounding of the pectoral muscles, on the left breast, in terfered with the use of the left arm for a long time, but at las accounts, that difficulty had vanished.

